# A highway crash risk assessment method based on traffic safety state division

**DOI:** 10.1371/journal.pone.0227609

**Published:** 2020-01-14

**Authors:** Dongye Sun, Yunfei Ai, Yunhua Sun, Liping Zhao

**Affiliations:** 1 China Transport Telecommunications & Information Center, Beijing China; 2 National Engineering Laboratory for Transportation Safety and Emergency informatics, Beijing, China; 3 Beijing Institute of New Technology Applications, Beijing, China; Tongii University, CHINA

## Abstract

In order to quantitatively analyze the influence of different traffic conditions on highway crash risk, a method of crash risk assessment based on traffic safety state division is proposed in this paper. Firstly, the highway crash data and corresponding traffic data of upstream and downstream are extracted and processed by using the matched case-control method to exclude the influence of other factors on the model. Secondly, considering the weight of traffic volume, speed and occupancy, a multi-parameter fusion cluster method is applied to divide traffic safety state. In addition, the quantitative relationship between different traffic states and highway crash risk is analyzed by using Bayesian conditional logistic regression model. Finally, the results of case study show that different traffic safety conditions are in different crash risk levels. The highway traffic management department can improve the safety risk management level by focusing on the prevention and control of high-risk traffic safety conditions.

## Introduction

Highway Traffic Safety Performance Assessment is one of the important means to guarantee its safe and efficient operation. In recent years, many researchers [1,2] have proposed a variety of traffic crash risk assessment and prediction models to find the most relevant risk factors affecting highway crashes, and to help traffic managers make correct and effective highway traffic safety management strategies. Highway crash risk is generally regarded as the sum of crash frequency and crash injury severity. Therefore, crash risk assessment and prediction models are generally based on crash frequency, rate and severity. There are some researchers using hurdle models [3] and logit model [4] to modeling the crash frequency with traffic data and the unbalanced panel data [5]. And some researchers analyze the highway crash severity by using Bayesian spatial generalized ordered logit model [6]. Simultaneously, some researchers proposed the Bayesian spatial random parameters Tobit model to analyzing crash rates of roadway segments [7].Moreover, the Bayesian multivariate random-parameter Tobit model and spatio-temporal correlation model are incorporated to analyze the relationship between the crash rates with the injury severity [8,9]. Based on the study of accident frequency, accident rate and accident severity, some more crash risk assessment and analysis methods and models have been put forward [10–13].

In addition, relevant studies show that the macroscopic traffic flow state at the same location shows different dynamic characteristics before and after the traffic crash [14]. Golob et al. found that different traffic flow states would lead to different types of traffic accidents [15, 16]. But only traffic flow state data in the case of accidents are used in this study, ignoring the influence of normal traffic flow state on the model. Moreover, Xu et al. [17] found that traffic flow state and traffic crash risk have a strong correlation by analyzing traffic flow data in the case of crashes and no crashes in the same place. His research shows that the dynamic characteristics of different traffic flow conditions have different influences on the highway crash occurrence mechanism. However, most of the existing crash risk assessment models fail to take into account the dynamic relationship between traffic crash mechanism and different traffic flow states [18]. In addition, the current researches in this field mainly refer to the macroscopic traffic flow state classification standard to carry out the traffic state classification process. Several traffic flow state evaluation indexes such as traffic flow, speed and occupancy are mainly used to divide traffic status in these macroscopic traffic flow state division methods [19]. However, the differences between accident traffic flow and normal traffic flow are not fully considered in these methods. Therefore, the similarity of status indicators may lead to the wrong classification of data samples. In view of this problem, the index weight optimization method [20] is used in this study to determine the comprehensive evaluation index and classification standard for expressway traffic flow state division. In the meantime, relevant studies [21, 22] have shown that there is a strong correlation between the upstream and downstream traffic flow state and the crash risk. In consequence, the crash and non-crash traffic flow data samples on the upstream and downstream of the crash location are collected and matched in case-control sample structure for cluster analysis. According to the result of cluster analysis, the traffic safety state of highway is divided. And then, the Bayesian conditional logistic regression model is proposed to evaluate the highway crash risk under different traffic safety conditions. Finally, the relative level of highway crash risk under different traffic safety conditions can be estimated by comparing the ratio values.

The remainder of this paper is organized as follows: The study area and data survey are declared in section 2. Section 3 introduces the highway crash risk assessment method based on traffic safety state division, including traffic state partition method based on multi-parameter fusion clustering and the crash risk assessment method based on Bayesian logistic regression. Section 4 verifies the proposed method with the real data mentioned in section 2, and discussed the results in details; Section 5 puts forward the main conclusions.

## Study area and data survey

**Fig 1:**
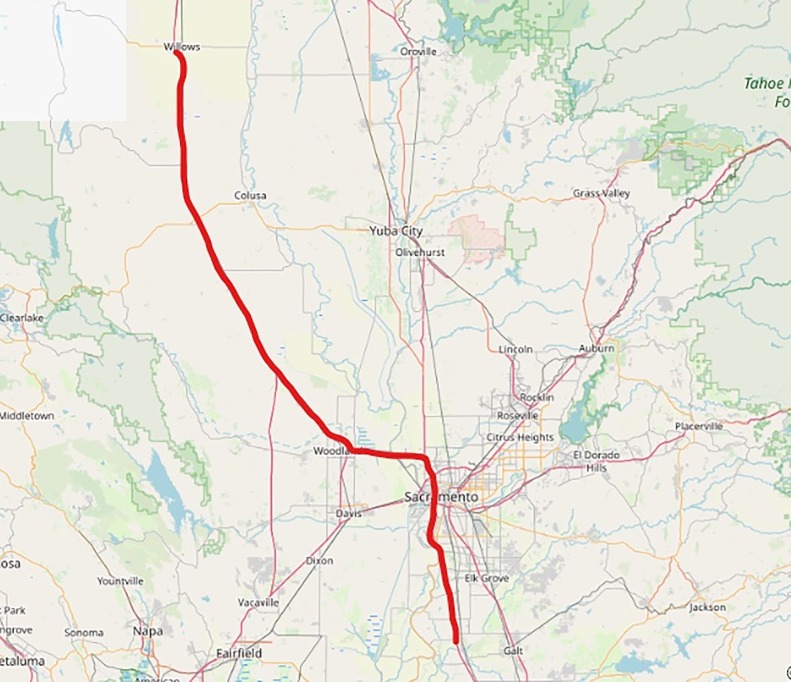
The specific location of interstate highway 5 in California, USA.

This study mainly focuses on the field of highway crash risk assessment. In order to achieve the above quantitative analysis and assessment between the traffic flow state and crash risk, it is necessary collecting a large number of highway crash data and corresponding traffic flow data. In view of the availability of the data needed in this research, all dataset was collected from the Performance Measurement System which can be freely download using URL: http://pems.dot.ca.gov/. In this research, the 45-mile section of interstate 5 in California, USA is selected as the research object (the absolute mileage pile number is 495.493–539.045 miles). The specific location is shown in the Fig 1. This map used in Fig 1 is rendered by Maperitive, which is a free image processing tool.

In order to exclude the influence of other influencing factors on the occurrence of traffic crashes, in the stage of data collection and processing, the matched case-control sample structure [23] is used for data matching. Where, case refers to the traffic flow state data when there is a traffic crash, and control refers to the traffic flow state data on the corresponding section when there is no traffic crash [24]. The basic principle of this data processing method is to verify the internal relationship between traffic flow state and traffic crash risk by comparing and analyzing the dynamic characteristics differences of traffic flow in the case of crashes and that in the case of no crashes.

**Fig 2:**
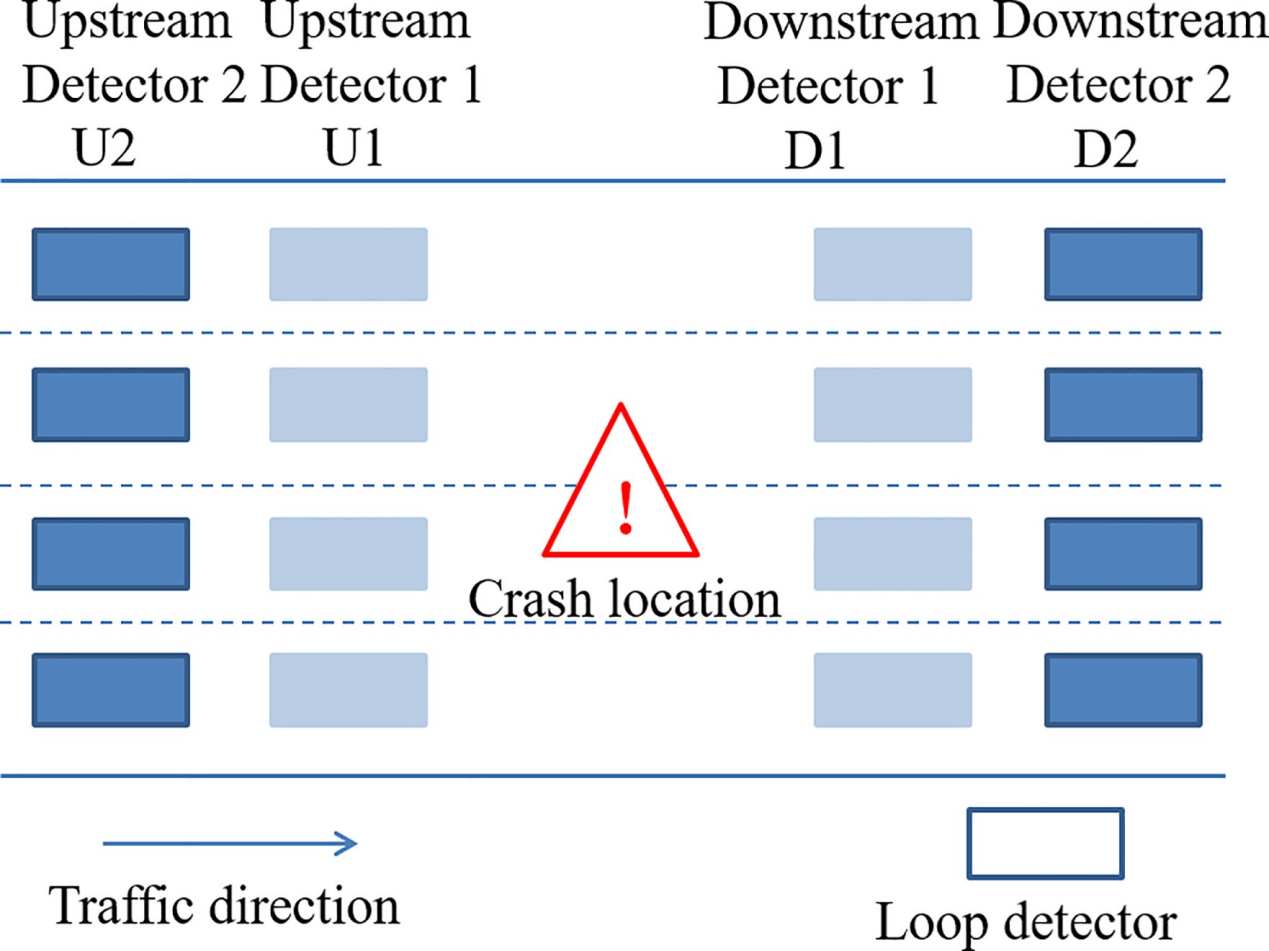
Sketch map of data extraction for traffic safety state analysis.

According to the requirements of the crash risk assessment model, traffic flow state data from 5min to 10min before the crash are used as the basic data for traffic safety state classification and crash risk assessment. Moreover, according to the matched case-control data processing method, the traffic flow status data of the four detectors in the upstream and downstream that are closest to the highway crash location are extracted. The upstream two detectors are named U2 and U1, while the downstream two detectors are named D2 and D1, as shown in Fig 2.

Because the raw data from the website includes three separate data sets, traffic flow dataset, crash dataset, and detector dataset. Therefore, based on the different types of datasets (traffic flow dataset, crash dataset, and detector dataset) are used in this study, the required database needs to be formed by matching different datasets according to the time and place attributes. First, according to the two attributes of the mileage pile number and the detector number in the detector dataset, the spatial correlation between the crash dataset and the traffic flow dataset was established to extract the traffic flow data (i.e. traffic volume, velocity, and occupancy) of the upstream and downstream detectors closest to the crash location. Then, according to the date and time attributes in crash dataset and traffic flow dataset, the temporal correlation between the crash data and traffic flow data was established to extract the 5min-10min traffic flow data before each crash time on the detector. Finally, according to the case-control matching principle, four groups of traffic flow status data without crash are extracted as the control by using the similar method. According to the data sample matching principle, 274 crash traffic flow data and 1096 non-crash traffic flow were correspondingly extracted in this study. Finally, a total of 1370 sets of data samples were obtained for highway crash risk modeling, in which the ratio of crash traffic flow to non-crash traffic flow was 1:4. The matched sample dataset is published in a public website. Interested researchers can download the three raw datasets (i.e. traffic flow dataset, crash dataset, and detector dataset) and the matched sample dataset using URL: https://doi.org/10.6084/m9.figshare.10303868.v1. And the matched sample dataset for highway crash risk evaluation is obtained by using the above data sample matching principle. In addition, it is confirmed that the authors did not have any special access privileges that others would not have.

## Methodologies

**Fig 3:**
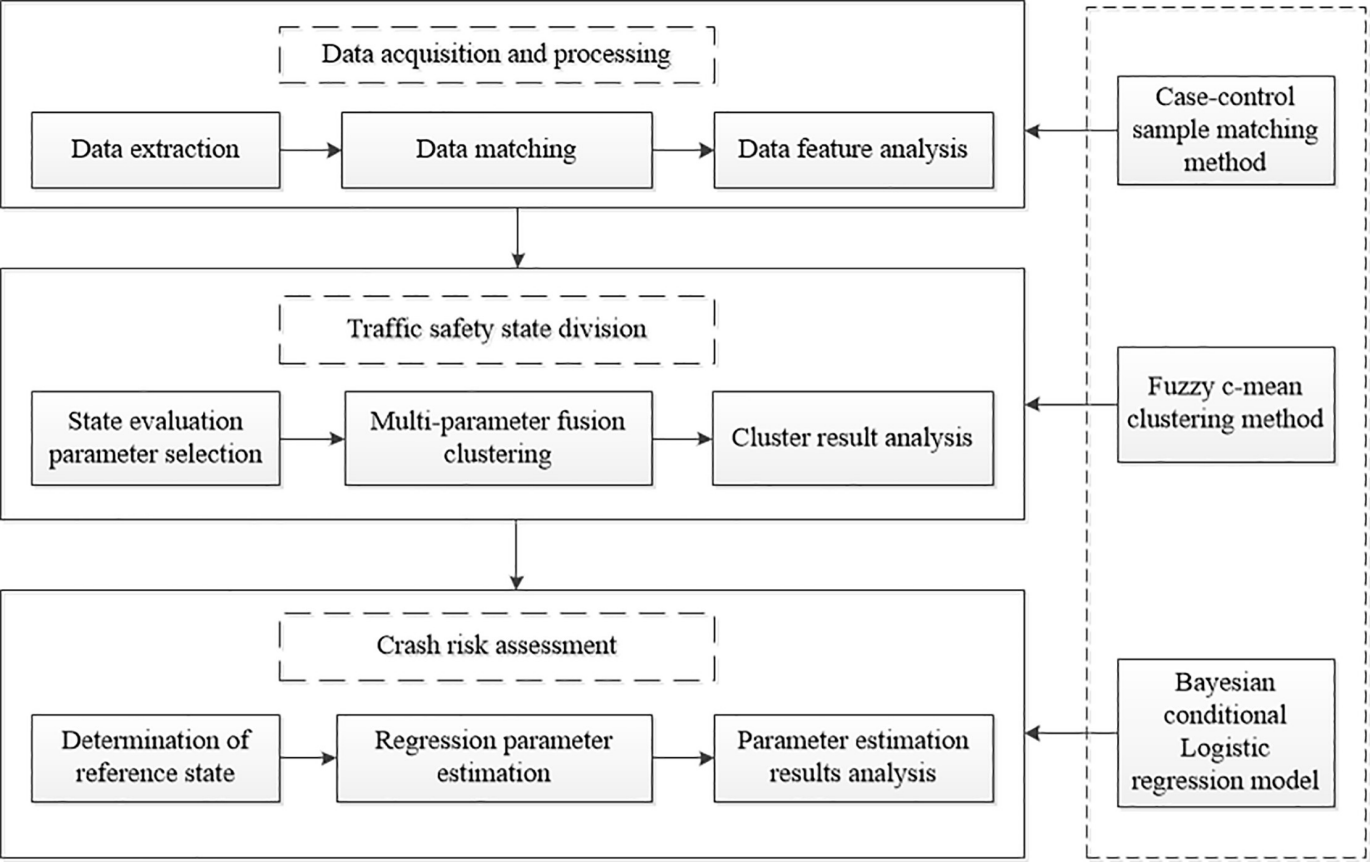
Flow chart of highway crash risk assessment method based on traffic state partition.

After applying the matched case-control method to match the sample data, multi-parameter fusion clustering method [25] is introduced to divide traffic safety states with the matched sample data. Thereafter, on the basis of the traffic safety status classification, the Bayesian conditional logistic regression model is put forward to evaluate the highway crash risk under different traffic safety conditions. The specific technical route of this research is shown in Fig 3.

### Traffic state partition method based on multi-parameter fusion clustering

Based on the fuzzy c-means cluster analysis method, the traffic state variables of the four detectors at the upstream and downstream of the crash location (i.e. U2, U1, D1, and D2) are used as the clustering index to classify the traffic safety state of the highway. Fuzzy c-mean (FCM) is a kind of clustering method based on objective optimization. This method minimizes the weighted distance sum of each sample to the fuzzy clustering center through iteration. And finally divides the data into c categories. Its optimization objective function is shown in the Eq 1.

$$\min\left\{J_{m}(U,v_{1},v_{2},\cdots ,v_{c})\right\}=\sum_{j=1}^{n}\sum_{i=1}^{c}{\left(\mu_{ij}\right)}^{m}{\left(d_{ij}\right)}^{2}$$ (1)

Where, *U* is the membership matrix of each data point and the corresponding clustering center, *v*_*c*_ is the *c*th fuzzy clustering center, *u*_*ij*_(*u*_*ij*_∈[0,1]) represents the membership degree of the *i*th data point belonging to the *j*th cluster center, *d*_*ij*_ is the Euclidean distance between the *i*th clustering center and the *j*th data point, *m*∈[1,∞] is a weighted index, with the increase of *m*, the fuzziness of clustering increases, and satisfies Eq 2.

$$\sum_{i=1}^{c}{\left(\mu_{ij}\right)}^{m}=1,\forall j=1,\cdots ,n.$$ (2)

The specific steps of the fuzzy c-means clustering method are shown below.

Step 1 Cluster category *c* is determined by using Eq 3, that is, the value of *c* that maximizes *L*. And the fuzzy coefficient *m* is generally set as 2. In addition, *ε* is the iteration stop threshold, and the maximum iteration number of the algorithm is *b*_*max*_. Initializes the membership matrix with random Numbers

$$L\left(c\right)=\frac{\sum_{i=1}^{c}\sum_{j=1}^{n}\mu_{ij}^{m}{\left\|v_{i}-\bar{x}\right\|}^{2}/\left(c-1\right)}{\sum_{i=1}^{c}\sum_{j=1}^{n}\mu_{ij}^{m}{\left\|x_{j}-v_{i}\right\|}^{2}/\left(n-c\right)}$$ (3)

Step 2 the fuzzy clustering center vector matrix ***V*** is calculated according to Eq 4.

$$v_{i}^{\left(b\right)}=\left[\sum_{j=1}^{n}{\left(\mu_{ij}^{\left(b\right)}\right)}^{m}\cdot x_{j}\right]/\left[\sum_{j=1}^{n}{\left(\mu_{ij}^{\left(b\right)}\right)}^{m}\right],\;i=1,2\cdots ,c$$ (4)

Step 3 the fuzzy clustering membership matrix *U* (b+1) is calculated and updated according to Eq 5.

$$\mu_{ij}^{\left(b+1\right)}={\left[\sum_{k=1}^{c}{\left(d_{ij}^{\left(b+1\right)}/d_{kj}^{\left(b+1\right)}\right)}^{2/\left(m-1\right)}\right]}^{-1},\;k=1,2\cdots ,c$$ (5)

Step 4 Compared the fuzzy clustering membership matrix *U*^(*b*)^ and *U*^(*b*+1)^. If ‖*U*^(*b*+1)^−*U*^(*b*)^‖≤*ε*, terminate the iteration. Otherwise, let *b* = *b*+1 go back to step 2, and continue to iterate until the maximum number of iterations is reached *b*_max_.

It should be noted that most existing studies use single evaluation indexes such as speed, flow rate or occupancy rate as input variables of fuzzy cluster analysis. In this paper, the traffic flow state evaluation index based on multi-parameter fusion is proposed as the input parameter of cluster analysis, which can not only ensure the effective division of traffic state, but also comprehensively reflect the traffic flow state information contained in multiple parameters.

### Crash risk assessment method based on Bayesian logistic regression

On the basis of traffic safety state division, the Bayesian conditional Logistic regression model is introduced to estimate the influence of different traffic safety states on expressway accident risk. In this model, one of the traffic safety states is taken as the control state. Then the ratio value of other traffic safety states to the control state is calculated respectively. And then the influence of different traffic safety states on crash risk is analyzed and evaluated according to the calculation results of the ratio value. The crash risk assessment model can be described as follows.

Suppose there are N matched case-control sample data, and in group j (j = 1, 2 …,N) contains 1 piece of crash traffic flow data and m pieces of normal traffic flow data. Therefore, for the *j*th group of data, its conditional likelihood is the probability of observation data in the premise of given observation number of all samples and crash sample number of matched samples. Let *p*_*j*_(**x**_ij_) is the probability that the *i*th piece traffic flow data in the *j*th group matched sample is crash traffic flow. Among them, **x**_ij_ = (*x*_1ij_,*x*_2ij_,…,*x*_kij_) is a vector composed of *K* traffic flow variables, and *i* = 0,1,2 … m; *j* = 1, 2, …N. Then the probability of crash occurrence can be expressed as a linear Logistic regression model, as shown in Eq 6.

$$\log\mathrm{it}\left[p_{j}\left(x_{ij}\right)\right]=\alpha_{j}+\beta_{1}x_{1ij}+\beta_{2}x_{2ij}+\cdots \cdots +\beta_{k}x_{kij}$$ (6)

Where, *α*_*j*_ represents the effect of matched variables on crash occurrence variables in the *j*th matched sample, which varies from the matched sample data. *β*_1_, *β*_2_,……,*β*_k_ represents the regression coefficient of explanatory variable. The conditional likelihood function is constructed to eliminate the sample bias caused by the paired sampling method.

It can be known **x**_0j_, **x**_1j_, …, **x**_mj_ are the *j*th matched explanatory variable from the above model. Under this condition, the conditional likelihood function of **x**_0j_ in the *j*th matched sample can be written as Eq 7.

$$P_{j}^{c}=\frac{p_{j}\left(x_{0j}|y=1\right)\prod_{i=1}^{m}\left(x_{ij}|y=0\right)}{\sum_{i=0}^{m}\left(x_{ij}|y=1\right)\prod_{i^{'}\neq i}^{}\left(x_{i^{'}j}|y=0\right)}=\frac{\exp\left(\sum_{k=1}^{K}\beta_{k}x_{k0j}\right)}{\exp\left(\sum_{k=1}^{K}\beta_{k}x_{k0j}\right)+\sum_{i=1}^{m}\exp\left(\sum_{k=1}^{K}\beta_{k}x_{kij}\right)}$$ (7)

Therefore, the likelihood function in conditional Logistic regression model can be expressed by Eq 8.

$$\begin{array}{l} f(Y|\beta )=\prod_{j=1}^{N}f\left(y_{0j}=1|\beta \right)=\prod_{j=1}^{N}P_{j}^{c}\\ \;=\exp\left\{\sum_{j=1}^{N}\sum_{k=1}^{K}\beta_{k}x_{k0j}-\sum_{j=1}^{N}\log\left[\sum_{i=0}^{N}\exp\left(\sum_{k=1}^{K}\beta_{k}x_{kij}\right)\right]\right\} \end{array}$$ (8)

The maximum likelihood estimation method is widely used in traffic safety modeling [26–27]. But this method belongs to point estimation and is difficult to estimate the parameters of this model. Markova Chain Monte Carlo method is proposed in this research to obtain the probability distribution of regression coefficient ***β***. It is applied to continuously sample from the posterior joint probability density distribution in order to generate parameter values randomly. As this distribution is not a standard distribution, the Metropolis-Hasting sampling method is adopted in this algorithm. The specific steps of the algorithm are as follows.

Step1 Let $\beta =(\beta_{0}^{\left(t-1\right)},\ldots ,\beta_{k}^{\left(t-1\right)})$
Step2 Generate candidate values from the proposed distribution **β**′ = (*β*_0_,…,*β_k_*)^*T*^
Step3 Calculate $\alpha =min\left(0,ln\frac{f(Y|\beta_{0}^{\prime },\ldots ,\beta_{k}^{\prime })f(\beta_{0}^{\prime },\ldots ,\beta_{k}^{\prime })}{f(Y|\beta_{0},\ldots ,\beta_{k})f(\beta_{0},\ldots ,\beta_{k})}\times \frac{N(\beta |\beta^{\prime })}{N(\beta^{\prime }|\beta )}\right)$
Step4 Randomly generate U from a uniform distribution of U(0,1)
Step5 If *ln*(*U*)≤*α*, then ***β***^***(t)***^ = ***β***^***’***^, otherwise ***β***^***(t)***^ = ***β***
Step6 Let t = t+1, go back to step 1

## Results and discussion

### Traffic safety state division

In this study, the input of FCM model is a data set with four characteristics, which is composed of the traffic state comprehensive evaluation index on the four detectors (i.e. U2, U1, D1, and D2) located upstream and downstream of the crash site. One output of FCM model is the fuzzy membership matrix *U* including *c* row and *n* column, which *c* is the clustering number and *n* is the number of data sample. The other output is the cluster center vector set *V*, which including *c* elements and each element is 4-dimensional.

Firstly, according to Eq 3, the final value of fuzzy clustering category *c* is determined. When *c* = 6, *L*(*c*) is the largest, so the traffic safety state is divided into 6 categories. Afterwards, according to the fuzzy c-means clustering steps, the fuzzy clustering center of highway data sample is obtained, as shown in Table 1.

**Table 1:** Fuzzy clustering center of 6 categories.

Name of input variable	State 1	State 2	State 3	State 4	State 5	State 6
comprehensive evaluation index of U2	22	33	46	33	26	36
comprehensive evaluation index of U1	20	32	47	38	23	32
comprehensive evaluation index of D1	22	32	37	61	36	28
comprehensive evaluation index of D2	21	33	34	49	31	28

According to the three-phase traffic flow theory, the traffic flow at a single detector can be divided into three traffic states (i.e. free flow, phase transition flow, and crowded flow). Theoretically, the four detectors have a total of 3^4^ = 81 combinations of traffic states. However, due to the close distance between detectors (about 0.3–0.8km), the traffic state of adjacent detectors on the upstream and downstream has a certain degree of similarity. In addition, there are obvious state differences between the matched crash and non-crash traffic flow sample data. As a result, the category number value obtained based on multi-parameter fusion clustering is significantly lower than the theoretical value. But it also indicates that only a few traffic conditions are more likely to cause highway traffic crash.

**Fig 4:**
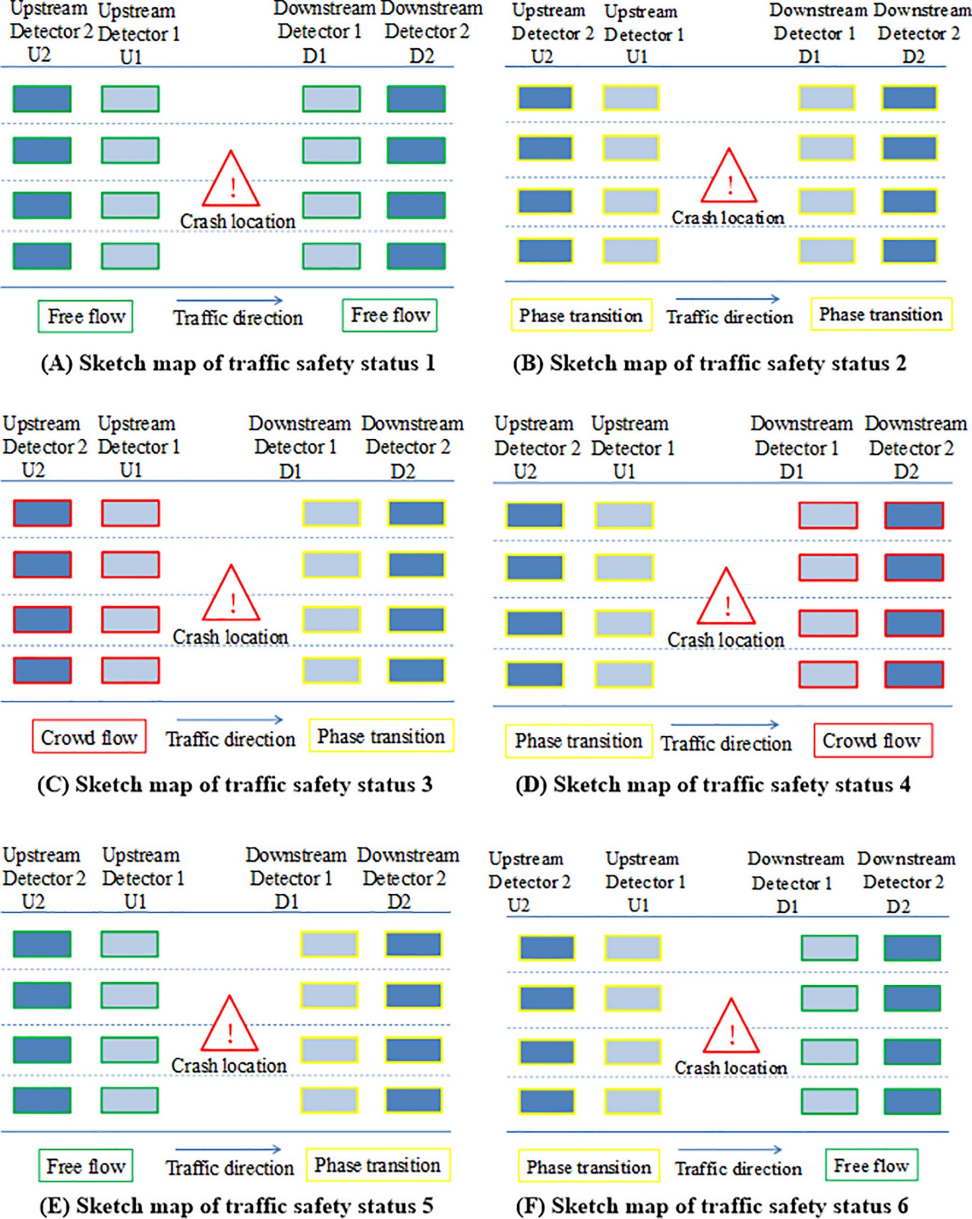
Characteristics of different traffic safety states. (A) Sketch map of traffic safety status 1. (B) Sketch map of traffic safety status 2. (C) Sketch map of traffic safety status 3. (D) Sketch map of traffic safety status 4. (E) Sketch map of traffic safety status 5. (F) Sketch map of traffic safety status 6.

According to clustering center of traffic safety state in Table 1 and the division of three-phase traffic flow, the six traffic safety states can be described with the corresponding upstream and downstream traffic flow characteristics. The schematic diagram of the six traffic safety states is shown in Fig 4.

As shown in Fig 4A, the traffic flow state of upstream and downstream is basically in the same condition. The comprehensive evaluation index of upstream traffic flow is 22 and 20 respectively, and the value of downstream traffic flow is 22 and 21 respectively. Traffic flow of upstream and downstream are both in free flow state. According to the sample clustering results, the proportion of non-crash samples in the total sample is 56.30%, the proportion of crash samples in the total sample is 54.38%, and the proportion of both in the total sample is 55.91%, indicating that more than half of the samples are in the traffic safety state 1. In addition, in this traffic safety state, the proportion of crash samples is 19.45% and that of non-crash samples is 80.55%. The proportion of crash samples and non-crash samples is close to 1:4.

As shown in Fig 4B, the traffic flow state of upstream and downstream is basically in the same condition. The comprehensive evaluation index of upstream traffic flow is 33 and 32 respectively, and the value of downstream traffic flow is 32 and 32 respectively. Traffic flow of upstream and downstream are both in phase transition flow state. According to the sample clustering results, the proportion of non-crash samples in the total sample is 8.85%, the proportion of crash samples in the total sample is 11.68%, and the proportion of both in the total sample is 9.42%, indicating that less than 10% of the samples are in traffic safety status 2. In addition, in this traffic safety state, the proportion of crash samples is 24.81% and that of non-crash samples is 75.19%. The proportion of crash samples and non-crash samples is close to 1:3.

As shown in Fig 4C, the traffic flow state of upstream and downstream is different. The comprehensive evaluation index of upstream traffic flow is 46 and 47 respectively, and the value of downstream traffic flow is 37 and 34 respectively. Traffic flow of upstream is in crowded state, and that of downstream is in phase transition state. According to the sample clustering results, the proportion of non-crash samples in the total sample is 6.48%, the proportion of crash samples in the total sample is 13.87%, and the proportion of both in the total sample is 7.96%, indicating that nearly 8% of the samples are in traffic safety status 3. In addition, in this traffic safety state, the proportion of crash samples is 34.86% and that of non-crash samples is 65.14%. The proportion of crash samples and non-crash samples is close to 1:2.

As shown in Fig 4D, the traffic flow state of upstream and downstream is quite different. The comprehensive evaluation index of upstream traffic flow is 33 and 38 respectively, and the value of downstream traffic flow is 61 and 69 respectively. Traffic flow of upstream is in phase transition state, and that of downstream is in crowded state. According to the sample clustering results, the proportion of non-crash samples in the total sample is 2.28%, the proportion of crash samples in the total sample is 4.01%, and the proportion of both in the total sample is 2.63%, indicating that less than 3% of the samples are in traffic safety status 4. In addition, in this traffic safety state, the proportion of crash samples is 30.56% and that of non-crash samples is 69.44%. The proportion of crash samples and non-crash samples is close to 2:5.

As shown in Fig 4E, the traffic flow state of upstream and downstream is different. The comprehensive evaluation index of upstream traffic flow is 26 and 23 respectively, and the value of downstream traffic flow is 36 and 31 respectively. Traffic flow of upstream is in free flow state, and that of downstream is in phase transition state. According to the sample clustering results, the proportion of non-crash samples in the total sample is 13.69%, the proportion of crash samples in the total sample is 6.93%, and the proportion of both in the total sample is 12.34%, indicating that 12% of the samples are in traffic safety status 5. In addition, in this traffic safety state, the proportion of crash samples is 11.24% and that of non-crash samples is 88.76%. The proportion of crash samples and non-crash samples is close to 1:8.

As shown in Fig 4F, the traffic flow state of upstream and downstream is different. The comprehensive evaluation index of upstream traffic flow is 36 and 32 respectively, and the value of downstream traffic flow is 28 and 28 respectively. Traffic flow of upstream is in phase transition state, and that of downstream is in free flow state. According to the sample clustering results, the proportion of non-crash samples in the total sample is 12.41%, the proportion of crash samples in the total sample is 9.12%, and the proportion of both in the total sample is 11.75%, indicating that less than 12% of the samples are in traffic safety status 6. In addition, in this traffic safety state, the proportion of crash samples is 15.53% and that of non-crash samples is 84.47%. The proportion of crash samples and non-crash samples is close to 1:5.

In addition, it should be pointed out that the sample proportions of crash traffic flow and non-crash traffic flow in the above six traffic safety states are different. According to the statistical analysis results, it can be found that only the sample proportion in traffic safety state 1 consistent with the original sample data (i.e. the ratio of crash samples to non-crash samples is 1:4). It indicates that only traffic safety state 1 has no sample deviation in the clustering process, while other safety states have sample deviation to some extent. At the same time, it also shows that only safety state 1 is less affected by the crash, while other states are more affected by the crash. In order to compare the different effect between six traffic safety states, further quantitative evaluation of several safety states is needed.

### Crash risk assessment

In order to further quantitatively analyze the relationship between six different traffic safety states and crash risk, the Bayesian conditional Logistic regression model was put forward to analyze the influence of different traffic safety states on the highway crash risk. It can be known that the traffic safety state has a strong correlation with traffic flow, speed, occupancy and other traffic state evaluation indicators. Therefore, only traffic safety state is considered as explanatory variables in conditional Logistic regression model. Moreover, discrete variables cannot be directly used as explanatory variables in the regression model. So, traffic safety state 1 (i.e. free flow state in upstream and downstream) is taken as the reference state in this study. And other traffic safety states are described by five parameters (i.e. ***β***_***1***_, ***β***_***2***_, ***β***_***3***_, ***β***_***4***_, ***β***_***5***_).

The Markova Chain Monte Carlo iteration process was implemented by applying WinBUGS14 in this study. And then the parameter value obtained from the least squares estimation was taken as the initial value of MCMC iteration. Finally, a Markova chain with 10,000 iterations was obtained based on which Bayesian inference was carried out. It is found that the model reached convergence after 4500 iterations, so the model parameters are inferred by using the values of the later 5500 iterations. The model estimation results are shown in Table 2.

**Table 2:** Estimation results of Bayesian conditional logistic regression model.

Explanatory variable	Parameter *β*	The mean of parameter	the standard deviation of parameter	Log-likelihood ratio Exp(*β*)
State 2	***β***_***1***_	1.586	0.299	4.884
State 3	***β***_***2***_	1.897	0.323	6.666
State 4	***β***_***3***_	2.089	0.365	8.077
State 5	***β***_***4***_	1.654	0.358	5.228
State 6	***β***_***5***_	1.438	0.372	4.212
State 1	—*	—	—	—

Note

* traffic safety state 1 is the reference state of other states

Log-likelihood ratio is an important parameter for evaluating conditional logistic regression models, which has many successful applications in the field of crash risk analysis and evaluation [28–29]. Since all the 95% confidence intervals for *β* in the model don't contain 0, it indicates that there are significant differences between these traffic safety states and reference state (i.e. traffic safety state 1). It can be seen from the estimated results of the model in Table 2, the log-likelihood ratios of the other traffic safety states are all greater than 1. It indicates that the crash risk level of the traffic safety state 1 is the lowest among the six traffic safety states. And the crash risk of the other traffic safety states is higher than that of the reference state. Moreover, more than half of the crash sample data are in traffic safety state 1, indicating that most of the highway traffic flow state is in free flow state, and the highway crash risk is relatively low.

In addition, it can be seen that the log-likelihood ratio of traffic safety state 4 is 8.077, which is the largest compared with other states. This indicates that the highway crash risk is the highest when the upstream is in a phase change flow state and the downstream is in a crowded flow state. This is mainly because the vehicle in the phase transition state has high speed and small traffic volume, while the vehicle in the crowded state has low speed and large traffic volume. When the vehicle operating environment suddenly changes from the phase transition state to the crowded state, traffic crashes are easy to occur, that is, there is a larger risk of crashes, which is consistent with the actual safety situation of the highway. Meanwhile, crash data samples in traffic safety state 4 only account for 2.63% in the total sample. This is in line with the occasional characteristics of highway traffic crashes.

Finally, according to the log-likelihood ratio, it can rank the crash risk levels in different states. The higher the log-likelihood ratio is, the greater the corresponding crash risk will be, and the greater the probability of crashes will be in this state. It can be seen that the corresponding crash risk is the highest in state 4 and state 3. These two can be regarded as the most dangerous traffic states, so as to help the highway management department to formulate preventive measures, reduce the crash rate and improve the safety management level.

## Conclusions

Highway crash risk assessment is one of the core tasks of highway traffic safety management and control. A based on traffic state division highway crash risk assessment method is proposed in this research. First, the case-control sample structure is applied to match the crash traffic flow data and non-crash traffic flow data. Secondly, the multi-parameter fusion evaluation index is taken as the clustering parameter for traffic state division. And then to apply the fuzzy c-means clustering method to classify the traffic safety state with the sample data. Finally, Bayesian conditional logistic regression model is proposed to evaluate the influence of different traffic states on highway crash risk. The result of case study shows that the crash risk level is different in different traffic states. Therefore, the highway safety management department should focus on strengthening the control measures of traffic state in high crash risk.
